# A Comparative Study on the Phytochemical Composition, Antioxidant, and Anti‐Inflammatory Effects of Stem Lettuce (*Lactuca sativa* var. *angustana*) Depending on the Presence of the Peel

**DOI:** 10.1002/fsn3.71207

**Published:** 2025-11-27

**Authors:** Sun Young Han, Ju Hong Park, Nami Joo

**Affiliations:** ^1^ Department of Food and Nutrition College of Human Ecology, Sookmyung Women's University Seoul Republic of Korea; ^2^ Department of Convergence IT Engineering Pohang University of Science and Technology (POSTECH) Pohang Republic of Korea

**Keywords:** anti‐inflammatory effect, antioxidant activity, phytochemicals, stem lettuce peel

## Abstract

With increasing interest in food sustainability and waste valorization, plant‐based by‐products have been explored as sources of functional compounds. Stem lettuce ( *Lactuca sativa* var. *angustana*), which is commonly consumed in East Asia, generates substantial peel waste that is largely underutilized. This study investigated the potential of stem lettuce peel as an upcycled functional food ingredient by evaluating its physicochemical characteristics, phytochemical profile, antioxidant activity, and anti‐inflammatory properties. Two sample types were prepared: unpeeled stem lettuce (USL) and peeled stem lettuce (PSL). The physicochemical characteristics were analyzed using conventional methods, and the phytochemicals were identified and quantified using UHPLC‐Triple TOF‐ESI‐MS/MS. Functional evaluations were conducted exclusively on USL and PSL samples, which were freeze‐dried and extracted using 70% ethanol. Antioxidant activity was assessed using total polyphenol and flavonoid content, and DPPH, ABTS, and ferric reducing antioxidant power (FRAP) assays. Anti‐inflammatory effects were tested in LPS‐stimulated RAW264.7, using the WST‐1 viability assay, nitric oxide (NO) production, and expression of inflammation‐related genes. USL showed significantly higher antioxidant activity and phenolic content than PSL. In total, 29 phytochemicals were identified, including hydroxycinnamic acids, flavonoids, and sesquiterpene lactones such as lactucopicrin and lactucin, predominantly in peel‐containing samples. USL markedly reduced NO production and inflammatory gene expression in a dose‐dependent manner, whereas PSL had minimal effects. These findings suggest that the stem lettuce peel has strong potential as a sustainable and functional ingredient for future food applications.

## Graphical abstract

This study evaluated stem lettuce ( *Lactuca sativa* var. *angustana* ) peels as functional food ingredients for sustainable waste valorization. Compared with peeled stem lettuce (PSL), unpeeled samples (USL) had higher phytochemicals, notably sesquiterpene lactones lactucin and lactucopicrin, greater total phenolics and flavonoids, stronger DPPH, ABTS, and FRAP antioxidant activity, and enhanced anti‐inflammatory effects by reducing nitric oxide and inflammatory gene expression in LPS‐stimulated RAW264.7 cells. Thus, stem lettuce peels represent a viable, sustainable, upcycled functional ingredient for future food applications. (d) A figure that best represents the scope of the paper. The image supplied should fit within the dimensions of 50 mm × 60 mm, and be fully legible at this size.

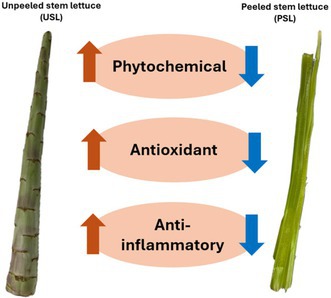

## 1 Introduction

The modern food industry is undergoing substantial expansion propelled by advancements in productivity and technology that prioritize sustainability, environmental stewardship, and resource recycling. The byproducts generated during food production and consumption have emerged as a significant global issue, and their annual volume is escalating. According to the Food and Agriculture Organization (FAO) of the United Nations, approximately one‐third of the global food supply, equating to approximately 1.3 billion tons, is wasted each year. This issue extends beyond food loss and contributes significantly to greenhouse gas emissions and environmental degradation (Galanakis 2012). Food upcycling has emerged as a promising strategy to mitigate these challenges by enhancing the value of low‐grade food byproducts and converting them into functional food ingredients, thereby optimizing resource utilization and reducing environmental impacts (Hrelia et al. 2023). Notably, plant‐based byproducts are often abundant in dietary fiber, phenolic compounds, flavonoids, and other bioactive substances, rendering them suitable for the development of functional foods (Llorach et al. 2008).

Stem lettuce ( *Lactuca sativa* var. *angustana*), a variety predominantly consumed in East Asia, is characterized by a thick stem, which is utilized in various culinary contexts because of its crunchy texture and flavor. This vegetable is rich in numerous bioactive compounds, particularly phenolic compounds, flavonoids, lactucins, lactucopicrin, sesquiterpene lactones, carotenoids, and both water‐soluble and fat‐soluble vitamins (B group, C, E) (Yang et al. 2022). A notable bioactive component, lactucopicrin, has been shown to inhibit the activation of the NF‐κB pathway in inflammatory macrophages and reduce inflammatory plaque accumulation in an animal model of atherosclerosis, thereby demonstrating anti‐inflammatory and cardiovascular protective properties (He et al. 2021).

During culinary preparation, the peel of the stem lettuce is typically discarded, resulting in a significant amount of byproducts. Historically, the peel has been largely overlooked, and its potential as a food ingredient has not been thoroughly investigated. Recent research has indicated that the stem lettuce peel contains bioactive polysaccharides that may exhibit immunomodulatory and antioxidant properties (Nie et al. 2022), suggesting that it can be utilized as a functional material rather than solely as waste. Moreover, natural antioxidants derived from plant sources are perceived as safer alternatives than their chemically synthesized counterparts, which aligns with consumer preferences (Hrelia et al. 2023). Antioxidants are known for their ability to alleviate oxidative stress by neutralizing reactive oxygen species in the body, thereby preventing cellular damage and providing antiaging, anticancer, and anti‐inflammatory benefits (Li et al. 2018). Consequently, plant‐based byproducts with significant antioxidant potential are increasingly being acknowledged not only as functional foods but also as sustainable food resources. This study aimed to analyze the bioactive components present in stem lettuce peel and investigate their functional effects by comparing the antioxidant and anti‐inflammatory activities of samples with and without the peel. In this study, we sought to elucidate the relationship between phytochemicals in stem lettuce peels and their functional properties and evaluate the potential of food byproducts as functional food ingredients.

This study hypothesizes that the peel of stem lettuce represents a primary reservoir of bioactive phytochemicals, and that its presence or removal exerts a substantial influence on the phytochemical composition as well as antioxidant and anti‐inflammatory activities. Moreover, the research aims to evaluate the potential of food‐processing by‐products as functional food ingredients by elucidating the mechanisms of action of these compounds, their interactions, their bioavailability in vivo, and their implications for human health.

## 2 Materials and Methods

### 2.1 Experimental Materials

Stem lettuce ( *Lactuca sativa* var. *angustana*) harvested in October 2024 from Wonju, Gangwon Special Self‐Governing Province (37.342220°N, 127.920158°E), was used as the experimental material. Stem lettuce was rinsed three times with running water, and excess moisture was removed. Some were utilized after peeling, whereas others were used without peeling. All the samples were freeze‐dried for 72 h using a freeze dryer (MCFD 8508; Operon Eng Co., Seoul, Korea), ground using a grinder (HB‐310; HiBell Co., Seoul, Korea), and sieved through a 100‐mesh standard sieve. The resulting powder was stored in a deep freezer (SF‐53U; NIHON FREEZER Co. Ltd., Tokyo, Japan) at −40°C for further analysis.

### 2.2 Proximate Composition Analysis

Proximate composition analysis of stem lettuce, including crude fat, crude protein, and ash content, was conducted according to the AOAC International (2019) methods. Moisture content was determined using the air‐drying method at atmospheric pressure in a drying oven (Cheil Science, Seoul, Korea) set to 105°C with a 5 g sample. Crude fat content was measured using an automatic fat extractor (Hydrotherm 13‐0031, Gerhardt, Königswinter, Germany) via ether extraction. The crude protein content was determined using an automatic nitrogen distillation apparatus (Kjeltec 8400TM; Foss Co., Hillerod, Denmark) following the micro‐Kjeldahl method. Ash content was analyzed using a muffle furnace (JSMF‐140T; SeojinAsan, Seoul, Korea) at 600°C. The carbohydrate content was calculated by subtracting the sum of the moisture, crude fat, crude protein, and ash contents from 100 g. Three samples were measured three times each, and the average value was reported.

### 2.3 Physicochemical Properties Analysis

#### 2.3.1 pH

The pH of stem lettuce was assessed by homogenizing a 5 g sample in 50 mL of distilled water, followed by measurement using a glass electrode pH meter F‐51 (HORIBA, Tokyo, Japan). Each of the two samples was measured three times, and the average value was reported.

#### 2.3.2 Salinity

For the evaluation of salinity, a 5 g sample was homogenized in 50 mL of distilled water, and salinity was measured using a salinity meter (ES‐421, ATAGO Co. Ltd., Tokyo, Japan). As with the pH, two samples were measured three times each, and the average value was reported.

#### 2.3.3 Soluble Solids Content

The soluble solid content, representing sugar concentration, was determined by homogenizing a 5 g sample with 50 mL of distilled water and measuring it using a refractometer (PAL‐1, ATAGO Co. Ltd., Tokyo, Japan). As with the other measurements, two samples were measured three times each, and the average value was reported.

### 2.4 Identification of Phytochemicals

Phytochemicals were extracted from freeze‐dried stem lettuce powder using a 70% ethanol solution. The extract was analyzed using the Ultimate 3000 ultra‐high‐performance liquid chromatography (UHPLC) system (Thermo Scientific, USA), employing a Waters Cortex T3 column (2.1 mm × 150 mm, 1.6 μm; Waters Co., Milford, MA, USA) for the separation of phytochemical constituents. The column was maintained at 45°C with a flow rate of 0.25 mL/min. The mobile phase consisted of distilled water (Solvent A) containing 0.1% formic acid and acetonitrile (Solvent B) containing 0.1% formic acid. The gradient elution protocol was as follows: 0–15 min, 97%–85% B; 15–50 min, 85%–100% B; 50–55 min, 100% B; 55–60 min, return to 97% B for re‐equilibration. Mass spectrometric analysis was conducted using a Triple TOF 5600+ system (AB Sciex, USA) in both positive and negative ion modes. MS1 and MS2 scans were acquired using the full‐scan and information‐dependent acquisition (IDA) modes. Electrospray Ionization (ESI) was performed with a scan range of 100–200 *m*/*z* for MS and 30–2000 *m*/*z* for MS/MS. Instrument conditions included nebulizing and heating gases (Ion Source 1 and 2) at 50 psi, curtain gas at 25 psi, and a desolvation temperature of 500°C. Ion spray voltages were +5.5 kV (positive mode) and −4.5 kV (negative mode), with Declustering Potentials of ±60 V. Collision Energy (CE) was ±10 eV, and Collision Energy Spread (CES) was ±35 ± 15 eV, using nitrogen (N_2_) as the collision gas. Phytochemicals were tentatively identified by comparing their mass spectra with those of reference libraries, including Scaffold Elements 2.2.1, MoNA (MassBank of North America), and NIST. The identification was based on accurate mass, isotope distribution, and characteristic fragmentation patterns.

### 2.5 Quantification of Phytochemicals

Phytochemicals were extracted from freeze‐dried stem lettuce powder using 70% ethanol. The resulting extract was analyzed using a Thermo Vanquish system (Thermo Fisher Scientific Inc., USA). Chromatographic separation was performed with a Cortecs C18 column (2.1 × 150 mm, 1.6 μm; Waters Co., Milford, MA, USA), maintained at 45°C, with a flow rate of 0.25 mL/min and an injection volume of 1 μL. The mobile phase was comprised of Solvent A (distilled water with 0.1% formic acid) and Solvent B (methanol/acetonitrile = 50:50, v/v, with 0.1% formic acid). The gradient elution program was as follows: 0–0.1 min, 5% B; 0.1–1.0 min, 5%–25% B; 1.0–10.0 min, 25%–95% B; 10.0–11.0 min, 95% B, followed by 4 min re‐equilibration to initial conditions. Mass analysis was performed using a Thermo TSQ Altis triple‐quadrupole mass spectrometer (Thermo Fisher Scientific Inc., Waltham, MA, USA) equipped with a heated electrospray ionization (H‐ESI) source. Ionization was carried out in static mode with spray voltages of +3500 V (positive mode) and −2500 V (negative mode). The gas settings were as follows: sheath gas, 50 arb; auxiliary gas, 10 arb; sweep gas, 1 arb. The ion transfer tube temperature was 325°C, and the vaporization temperature was 350°C.

### 2.6 Analysis of Antioxidant Activity

#### 2.6.1 Preparation of Extracts

Peeled stem lettuce (PSL) and unpeeled stem lettuce (USL) samples were freeze‐dried and ground into powder. Each 10 g sample was mixed with 100 mL of 70% ethanol (Merck, Darmstadt, Germany) and extracted in a shaking incubator (SI‐900R; Jeio Tech, Daejeon, Korea) at 25°C and 250 rpm for 24 h. The extracts were filtered through No. 4 filter paper (Whatman Inc., Maidstone, UK) and used for antioxidant activity analysis.

#### 2.6.2 Total Polyphenol Content

Total polyphenol content was measured using the modified Folin–Ciocalteu method (Singleton et al. 1999). In a 50 mL test tube, 0.5 mL of each extract was mixed with 2.5 mL of 2N Folin–Ciocalteu reagent and 7.5 mL of 20% sodium carbonate (Na_2_CO_3_). The final volume was adjusted with distilled water, and the mixture was incubated in the dark for 2 h. The absorbance was measured at 765 nm using a spectrophotometer (T60UV, PG Instruments Ltd., Lutterworth, England). Gallic acid (Sigma‐Aldrich, St. Louis, MO, USA) was used as a standard, and the samples were analyzed in the same manner. Results were expressed as μg gallic acid equivalent (GAE, dry basis) per gram of extract based on the calibration curve.

#### 2.6.3 Total Flavonoid Content

The total flavonoid content was measured using a modified aluminum chloride colorimetric method (Chang et al. 2002). The reaction mixture was prepared using 2 mL of each extract, 2 mL of 10% aluminum chloride, 2 mL of 1 M potassium acetate, and 11.2 mL of distilled water. The mixture was incubated in the dark for 30 min, and the absorbance was measured at 415 nm using a spectrophotometer. Quercetin (Sigma‐Aldrich, St. Louis, USA) was used as a standard, and the samples were analyzed in the same way. Results were expressed as μg quercetin equivalent (CE, dry basis) per gram of extract, based on the calibration curve.

#### 2.6.4 DPPH Radical Scavenging Activity

The DPPH radical scavenging activity was measured by modifying the method of Im et al. (2008). The experimental group consisted of 0.2 mL of each extract mixed with 1.8 mL of 0.4 mM DPPH solution, while the control group consisted of 0.2 mL of 99.99% ethanol mixed with 1.8 mL of 0.4 mM DPPH solution. A color correction group was prepared by mixing 0.2 mL of each extract with 1.8 mL of 99.99% ethanol. All mixtures were incubated in the dark at room temperature for 30 min, and the absorbance was measured at 517 nm. The absorbance of the experimental group was corrected by subtracting the absorbance of the color‐corrected group, and the radical scavenging activity was calculated using the following formula:

$$\text{DPPH radical scavenging activity}\;\left(\%\right)=\left(1–A/B\right)\times 100$$

*A*, absorbance of the experimental group; *B*, absorbance of the control group.

#### 2.6.5 ABTS Radical Scavenging Activity

The ABTS^+^ radical scavenging activity was measured by modifying the method described by Re et al. (1999). A 1 mL solution of 7 mM ABTS was mixed with 1 mL of 2.45 mM potassium persulfate solution and kept in the dark for 16 h to generate ABTS^+^ radicals. Absorbance of the radical solution was measured at 734 nm and adjusted to 0.70 ± 0.02. A mixture of 1980 μL of the ABTS^+^ solution and 20 μL of the extract was incubated in the dark for 6 min, and the absorbance was measured at 734 nm. Trolox (Sigma‐Aldrich, St. Louis, MO, USA) was used as the standard, and the samples were analyzed in the same manner. The results are expressed as mM Trolox per gram of extract based on the calibration curve.

#### 2.6.6 Ferric Reducing Antioxidant Power (FRAP)

The FRAP was measured by modifying the method described by Oyaizu (1986). A mixture of 1 mL of extract, 2.5 mL of 0.2 M sodium phosphate buffer (pH 6.6), and 2.5 mL of 1% potassium ferrocyanide was incubated in a water bath at 50°C for 20 min. After incubation, 10% trichloroacetic acid (2.5 mL) was added, and the mixture was centrifuged at 3000 rpm for 10 min. From the supernatant, 2.5 mL was mixed with 2.5 mL of distilled water and 0.5 mL of 0.1% ferric chloride solution. Absorbance was measured at 700 nm. Ascorbic acid (Sigma‐Aldrich, St. Louis, MO, USA) was used as a standard, and the samples were analyzed in the same manner. The results were expressed as mg ascorbic acid equivalent (AAE) per gram of extract based on the calibration curve.

### 2.7 Analysis of Anti‐Inflammatory Activity

#### 2.7.1 Preparation and Treatment of Extracts

PSL and USL samples were used to evaluate anti‐inflammatory properties. After freeze‐drying, 10 g of each powdered sample was mixed with 100 mL of 70% ethanol and extracted by shaking in an incubator (SI‐900R; Jeio Tech, Daejeon, Korea) at 25°C and 250 rpm for 24 h. The resulting extracts were filtered through No. 4 filter paper (Whatman Inc., Maidstone, UK), concentrated under reduced pressure using a rotary evaporator (N‐1000; Eyela, Tokyo, Japan), and freeze‐dried for 72 h using a freeze‐dryer (MCFD 8508, Operon Eng Co., Seoul, Korea). The dried extracts were ground using a grinder (HB‐310; HiBell Co., Seoul, Korea) and passed through a 100‐mesh standard sieve. The final powdered samples were reconstituted in 80% ethanol to a final concentration of 0.5 mg/mL, and the resulting stock solutions were used for cytotoxicity evaluation, nitric oxide (NO) assays, and analysis of inflammation‐related gene expression.

#### 2.7.2 Cell Viability Assay (WST‐1)

Cytotoxicity was evaluated using the WST‐1 assay in Raw264.7 macrophages obtained from the American Type Culture Collection (ATCC). Cells were cultured in Dulbecco's modified Eagle's medium (DMEM) supplemented with 10% fetal bovine serum and 1% antibiotics/antimycotics and maintained at 37°C in a humidified atmosphere containing 5% CO₂. Cells were seeded in 96‐well plates at a density of 8 × 10 cells/well and incubated for 24 h. After removing the medium, samples were diluted to concentrations of 1.00, 0.50, 0.25, 0.13, 0.06, 0.03, 0.02, and 0.01 mg/mL, and 100 μL of each dilution was added to the wells. After 24 h of treatment, the cell viability was measured using the WST‐1 assay. The negative control (NC) was treated with medium only, and the positive control (PC) was treated with 10 μM lactucopicrin.

#### 2.7.3 Inhibition of Nitric Oxide Production

NO production was assessed using the Griess Reagent System (Promega). Raw264.7 cells were cultured in a 6‐well plate at a density of 8 × 10^3^ cells per well and incubated for 24 h to ensure optimal cell adhesion. Following the removal of the culture medium, lipopolysaccharide (LPS) was introduced at a concentration of 1 μg/mL, either alone or in combination with the test samples, which were prepared at concentrations of 1.0, 0.5, and 0.1 mg/mL. LPS was sourced from a 1 mg/mL stock solution that was diluted 1000‐fold, whereas the test samples were also prepared as 100× stock solutions prior to their addition to LPS and subsequent application to the cells. After a 24‐h treatment period, the supernatant was collected and absorbance was measured at 540 nm in accordance with the Griess reaction protocol. The measurements were performed using a SpectraMax ABS apparatus.

#### 2.7.4 Analysis of Inflammatory Gene Expression

The expression levels of anti‐inflammatory genes were assessed using quantitative reverse transcription polymerase chain reaction (qRT‐PCR). Following the experimental procedures, cell pellets were preserved at −80°C, and RNA was extracted using the RNeasy Mini Kit (QIAGEN). The concentration of RNA was determined by measuring absorbance at 260 and 280 nm, with only those samples exhibiting an OD_260_/_280_ ratio of 1.8 or greater being included in the subsequent analysis. Total RNA was then diluted with RNase‐free water to a concentration of 100 ng/μL, which was subsequently used for the synthesis of complementary DNA (cDNA). The resulting cDNA was analyzed by qPCR using SYBR Green PCR Master Mix (Enzynomics). Gene expression levels were quantified using glyceraldehyde‐3‐phosphate dehydrogenase (GAPDH) as an endogenous control. The qPCR protocol included an initial denaturation step at 95°C for 10 min, followed by 40 cycles consisting of denaturation at 95°C for 15 s, annealing at 58°C for 20 s, and extension at 72°C for 30 s.

### 2.8 Statistical Analysis

Statistical analysis was performed using SPSS software (Version 26, IBM Corporation, Armonk, NY, USA). One‐way analysis of variance (ANOVA) was used to assess differences among multiple groups, and an independent *t*‐test was used to compare two groups. Duncan's multiple range test was used to determine the significance of the means, with a significance threshold of *p* < 0.05. All experiments were conducted in triplicate (*n* = 3).

## 3 Results and Discussion

### 3.1 Proximate Composition

The results of the proximate composition analysis of stem lettuce are summarized in Table 1. The caloric value of stem lettuce was determined to be 71.7 kJ (17 kcal), which is lower than the caloric values of crisphead lettuce (80.07 kJ (20.84 kcal); Negrao et al. 2020), romaine lettuce (87.9 kJ (21 kcal)), and leaf lettuce (92 kJ (22 kcal); U.S. Department of Agriculture (USDA) 2022). The moisture content was 94.93%, which is comparable to that of crisphead lettuce (94.82%) (Negrao et al. 2020), romaine lettuce (94.3%), and leaf lettuce (94.0%) (USDA 2022). The crude protein content was 0.63%, which was lower than that found in crisphead lettuce (1.45%; Negrao et al. 2020), romaine lettuce (0.98%), and leaf lettuce (1.09%; USDA 2022). The ash content was 0.83%, which is lower than that of crisphead lettuce (0.96%; Negrao et al. 2020), yet higher than that of romaine lettuce (0.61%) and leaf lettuce (0.67%; USDA 2022). The carbohydrate content was 3.6%, which surpasses that of crisphead lettuce (1.97%; Negrao et al. 2020), but is lower than that of romaine lettuce (4.06%) and leaf lettuce (4.07%; USDA 2022). These differences in proximate composition may be associated with varietal characteristics and cultivation conditions, which can influence nutrient accumulation in lettuce species.

**TABLE 1:** Proximate composition of raw *Lactuca sativa* var. *angustana*.

Proximate compostion	Contents (g)
Moisture	94.93 ± 0.23
Carbohydrate	3.60 ± 0.20
Crude fat	0.00 ± 0.00
Crude protein	0.63 ± 0.06
Crude ash	0.83 ± 0.06

*Note:* Values are mean ± SD (*n* = 3).

### 3.2 Physicochemical Properties

#### 3.2.1 pH

The pH data are presented in Table 2. USL exhibited a pH of 6.21, whereas PSL showed a pH of 6.20. Statistical analysis indicated no significant difference between the two groups (*p* > 0.05), suggesting that peel treatment does not markedly affect the pH of stem lettuce.

**TABLE 2:** Physicochemical properties of stem lettuce depending on the presence of peels.

	PSL	USL
pH	6.20 ± 0.01	6.21 ± 0.01
Salinity (%)	0.5 ± 0.00	0.6 ± 0.00
Brix degree (°Brix)	0.4 ± 0.00	0.4 ± 0.00

*Note:* Values are expressed as mean ± SD (*n* = 3). No significant differences were observed between groups (*p* > 0.05).

Abbreviations: PSL, peeled stem lettuce; USL, unpeeled stem lettuce.

#### 3.2.2 Salinity

The salinity measurements are summarized in Table 2. The USL sample exhibited a salinity of 0.6, whereas the PSL sample exhibited a slightly lower salinity of 0.5. However, the differences in salinity between the two samples were not statistically significant (*p* > 0.05), indicating that the peel treatment may have a minimal effect on the salinity of stem lettuce. This suggests that there was no considerable variation in the inorganic ion content between the outer and inner tissues of stem lettuce. These results imply that peel removal does not markedly alter ion distribution, which is consistent with the stable allocation of inorganic compounds during plant growth.

#### 3.2.3 Soluble Solids Content

The soluble solid content data are presented in Table 2. All samples exhibited a uniform soluble solid content of 0.4, with no significant differences attributable to the peel treatment (*p* > 0.05). This consistency implied that the total soluble solids were similarly distributed between the peel and inner tissues of the stem lettuce. Soluble solids, primarily sugars, function as energy storage compounds and are typically distributed evenly throughout the stem and leaf tissues during plant development, suggesting no preferential accumulation in the peel region. Previous studies have also reported that soluble sugars in leafy vegetables do not differ significantly between the epidermal and central tissues (Kang and Saltveit 2002), and the results of the present study corroborate these findings. This further supports the distribution of soluble sugars that is generally uniform during plant development, regardless of peel removal.

### 3.3 Phytochemical Identification

**FIGURE 1:**
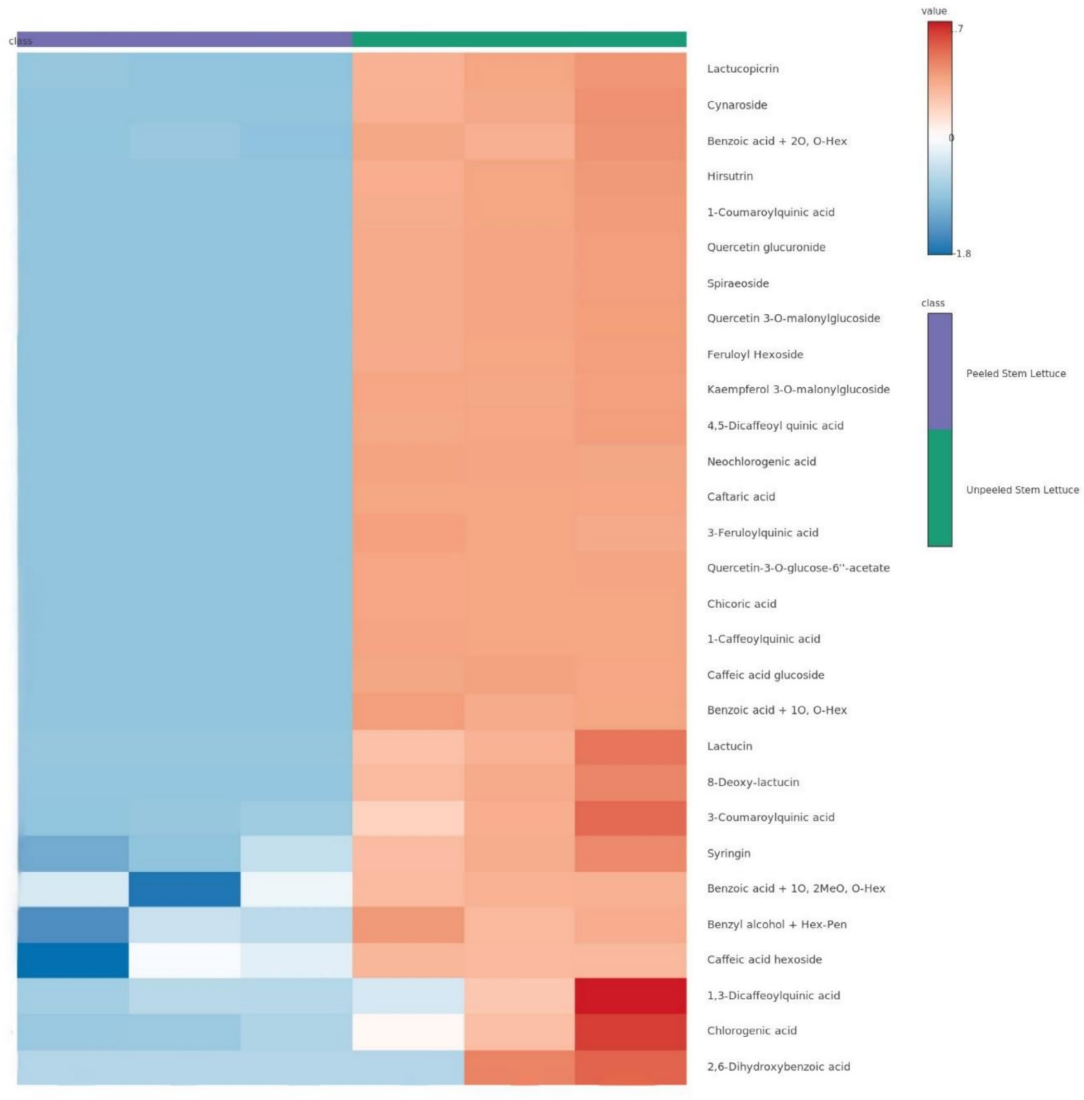
Heatmap of the phytochemicals of stem lettuce depending on the presence of peels. Heatmap of phytochemicals in peeled and unpeeled stem lettuce. Color intensity (red = higher, blue = lower) represents log_10_ intensity. Each column indicates a replicate sample, and each row a compound.

Qualitative assessment of the phytochemicals was performed using the UHPLC‐Triple TOF‐ESI MS/MS system in both positive and negative ionization modes. The results of this analysis are presented in Table 3 and indicate the tentative identification of 29 distinct phytochemicals. These compounds were classified according to their structural characteristics into five types of hydroxybenzoic acids, 13 types of hydroxycinnamic acids, one type of lignan, seven types of flavonoids, and three types of sesquiterpenes. These results indicated that the peel of stem lettuce contains a diverse array of phenolic compounds, flavonoids, and sesquiterpenes. The presence and intensity of these compounds were markedly higher in samples containing peel, suggesting that the outer tissue serves as a primary reservoir for bioactive compounds. A comparative analysis of the detection patterns across the samples revealed that only nine phytochemicals were present in the PSL sample, exhibiting overall lower ion intensities than those in the USL sample. In contrast, the USL sample demonstrated a broader array of compounds and higher detection intensities for most of the components. Figure 1 illustrates the differences in phytochemical composition among the three samples via heatmap visualization, reinforcing the observation that phytochemicals were more abundantly distributed in samples containing the peel. These findings imply that lettuce stem peels may function as a primary reservoir of phytochemicals.

**TABLE 3:** Identification of the phytochemicals of stem lettuce depending on the presence of peels.

	Analyte name	R.T. (min)	Molecular formula	Molecular weight	Adduct	*m*/*z*	Log_10_Precursor intensity	
							PSL	USL
**Hydroxybenzoic acid**								
1	Benzoic acid +1O, O‐Hex	4.17	C_13_H_16_O_8_	300.0845175	[M−H]−	299.078	—	4.497015
2	Benzoic acid +2O, O‐Hex	5.12	C_13_H_16_O_9_	316.0794321	[M−H]−	315.074	6.154740	6.530105
3	2,6‐Dihydroxybenzoic acid	6.68	C_7_H_6_O_4_	154.0266087	[M+H]+	155.033	—	4.252469
4	Benzoic acid +1O, 2MeO, O‐Hex	7.67	C_15_H_2_0O_10_	360.1056468	[M−H]−	359.099	3.357209	5.145013
5	Benzyl alcohol + Hex‐Pen	13.09	C _18_H _26_O _10_	402.152597	[M+HCO_2_]−	447.152	4.308569	5.085093
**Hydroxycinnamic acid**								
1	Caftaric acid	6.74	C_13_H_12_O_9_	312.048132	[M−H]−	311.041	—	5.229723
2	Neochlorogenic acid	7.37	C_16_H_18_O_9_	354.0950822	[M−H]−	353.088	—	4.908886
3	Caffeic acid hexoside	8.23	C_15_H_18_O_9_	342.0950822	[M−H]−	341.089	3.722525	5.282358
4	Caffeic acid glucoside	10.18	C_15_H_18_O_9_	342.0950822	[M−H]−	341.089	—	4.882414
5	Chlorogenic acid	10.75	C_16_H_18_O_9_	354.0950822	[M+Na]+	377.084	5.847117	6.103551
6	Feruloyl Hexoside	12.90	C_16_H_18_O_9_	356.1107322	[M−H]−	355.104	—	4.227088
7	1‐Caffeoylquinic acid	13.25	C_16_H_18_O_8_	354.0950822	[M−H]−	353.089	—	5.245875
8	3‐Coumaroylquinic acid	13.72	C_16_H_18_O_8_	338.1001675	[M+H]+	339.108	4.779710	5.379349
9	3‐Feruloylquinic acid	15.67	C _17_H _20_O _9_	368.1107322	[M−H]−	367.104	—	4.700319
10	1‐Coumaroylquinic acid	16.16	C_16_H_18_O_8_	338.1001675	[M+H]+	339.108	—	5.227550
11	Chicoric acid	17.97	C_22_H_18_O_12_	474.079826	[M−H]−	473.074	—	6.515713
12	1,3‐Dicaffeoylquinic acid	21.07	C_25_H_24_O_12_	516.1267762	[M+H]+	517.135	5.810772	6.008286
13	4,5‐Dicaffeoyl quinic acid	21.89	C_25_H_24_O_12_	516.1267762	[M−H]−	515.121	—	4.336611
**Lignan**								
1	Syringin	11.66	C_17_H_24_O_9_	372.1420	[M+Na]+	395.131	3.933568	4.779443
**Flavonoids**								
1	Spiraeoside	18.77	C_21_H_2_0O_12_	464.0954761	[M+H]+	465.104	—	5.661886
2	Quercetin glucuronide	19.65	C_21_H_18_O_13_	478.0747406	[M+H]+	479.083	—	5.507336
3	Hirsutrin	19.85	C_21_H_2_0O_12_	464.0954761	[M+H]+	465.103	—	4.882814
4	Cynaroside	20.1	C_21_H_2_0O_11_	448.1005615	[M+H]+	449.108	—	4.569267
5	Quercetin 3‐O‐malonylglucoside	20.67	C_24_H_22_O_15_	550.09587	[M+Na]+	573.085	—	6.114346
6	Quercetin‐3‐O‐glucose‐6″‐acetate	20.67	C_23_H_22_O_13_	506.1060	[M−H]−	505.101	—	6.092930
7	Kaempferol 3‐O‐malonylglucoside	22.33	C_24_H_22_O_14_	534.1010	[M−H]−	533.096	—	4.940926
**Sesquiterpenes**								
1	8‐Deoxy‐lactucin	12.32	C_15_H_16_O_4_	260.1049	[M+H]+	266.112	—	3.850040
2	Lactucin	13.19	C_15_H_16_O_5_	276.0998	[M+H−H_2_O]+	259.097	—	3.612911
3	Lactucopicrin	26.16	C_15_H_16_O_6_	410.1366	[M+H]+	411.145	4.824485	6.638431

*Note:* Analytes were identified based on retention time (R.T.), molecular formula, molecular weight, and mass spectral data (*m*/*z*, adduct). Values of precursor ion intensity are expressed on a log_10_ scale (*n* = 3).

Abbreviations: PSL, peeled stem lettuce; USL, unpeeled stem lettuce.

### 3.4 Phytochemical Quantification

The results of the quantitative analysis conducted using UHPLC‐Triple TOF‐ESI MS/MS are presented in Table 4. Fourteen major phytochemicals were quantified, including seven hydroxycinnamic acids, one lignan, two flavonoids, and two sesquiterpenes. The analysis revealed significant variations in the concentrations of most compounds among the samples (*p* < 0.001), with a consistent trend of elevated levels in the samples that included the peel. The flavonoid hirsutrin exhibited the highest concentration in the USL sample at 161.08 ± 1.99 μg/g, whereas it was markedly lower at 1.68 ± 0.10 μg/g in the PSL sample. Furthermore, the sesquiterpenes lactucin and lactucopicrin showed the most pronounced differences depending on the presence of the peel, with peak levels in the USL sample at 87.15 ± 4.45 μg/g and 618.37 ± 29.38 μg/g, respectively, in contrast to the PSL sample, where they were detected at 1.92 ± 0.16 μg/g and 5.04 ± 0.24 μg/g, reflecting over a 100‐fold difference. These results suggest that sesquiterpene phytochemicals are highly concentrated in the peel of stem lettuce, indicating their potential role in mediating anti‐inflammatory and antioxidant functions. The substantial decrease in these compounds following peel removal further supports the notion that the peel serves as a key phytochemical reservoir. Qualitative and quantitative analyses using UHPLC‐Triple TOF‐ESI MS/MS identified a diverse array of phytochemicals, particularly from the hydroxycinnamic acid, flavonoid, and sesquiterpene families, with notable concentrations of sesquiterpene compounds, such as lactucopicrin and lactucin, present in the peel samples. These higher concentrations in USL provide a biochemical bridge from composition to function, anticipating the stronger antioxidant and anti‐inflammatory phenotypes observed below.

**TABLE 4:** Quantification of the phytochemicals of stem lettuce depending on the presence of peels.

	Compounds	PSL	USL	*t*
**Hydroxycinnamic acid**				
1	Caftaric acid	2.64 ± 0.2	26.63 ± 2.99	−14.059***
2	Neochlorogenic acid	1.7 ± 0.09	7.53 ± 0.16	−43.392 ***
3	Chlorogenic acid	121.73 ± 9.8	262.25 ± 14.23	−11.032***
4	3‐Feruloylquinic acid	0.07 ± 0.01	0.31 ± 0.17	−23.768***
5	Chicoric acid	57.14 ± 0.71	218.46 ± 15.01	−19.532***
6	1,3‐Dicaffeoylquinic acid	34.55 ± 1.36	74.76 ± 2.45	−19.371***
7	4,5‐Dicaffeoylquinic acid	1.1 ± 0.75	4.08 ± 0.6	−43.527***
**Lignan**				
1	Syringin	23.58 ± 3.73	112.8 ± 13.6	−8.646***
**Flavonoids**				
1	Hirsutrin	1.68 ± 0.1	161.08 ± 1.99	−122.314***
2	Cynaroside	0.02 ± 0.00	5.82 ± 0.36	−24.534***
**Sesquiterpenes**				
1	Lactucin	1.92 ± 0.16	87.15 ± 4.45	−25.761***
2	Lactucopicrin	5.04 ± 0.24	618.37 ± 29.38	−40.202***

*Note:* Values are expressed as mean ± SD (*n* = 3). Statistical significance was determined by independent *t*‐test. Symbols indicate significance: *p* < 0.001 (***).

Abbreviations: PSL, peeled stem lettuce; USL, unpeeled stem lettuce.

### 3.5 Antioxidant Activity

#### 3.5.1 Total Polyphenol Content

Table 5 shows the total polyphenol content of stem lettuce, contingent upon peel treatment. The USL exhibited a total polyphenol content of 274.083 μg GAE/g, in contrast to the PSL, which demonstrated a significantly reduced level of 225.472 μg GAE/g. These findings suggest that polyphenolic phytochemicals are predominantly concentrated in the stem lettuce peel. Plant epidermal tissues accumulate various phenolic phytochemicals as defense mechanisms against environmental stressors that are intrinsically associated with antioxidant and antimicrobial properties (Zhou et al. 2016). Moreover, polyphenols are primarily located within the cell wall or cortex, indicating that the removal of the peel leads to the loss of these tissues and a subsequent decline in the total polyphenol content. The results of this study indicate that the peel of stem lettuce is a significant source of phytochemicals. Prior research has reported that the total polyphenol content in romaine lettuce is 203.5 μg GAE/g, while the polyphenol levels in stem lettuce samples with peel are comparatively elevated (Kim et al. 2019). The antioxidant properties observed in this study are ascribed mainly to polyphenols and flavonoids, which act through hydrogen atom or electron donation and chelation of transition metal ions (Andrés et al. 2023; Lakey‐Beitia et al. 2021). Flavonoids, in particular, are well documented for stabilizing free radicals via a hydrogen atom transfer (HAT) mechanism (Hassanpour and Doroudi 2023; Vuzem and Pilepić 2025). Consistent with these mechanisms, the higher total polyphenol and flavonoid levels measured in USL provide a direct chemical rationale for its superior antioxidant activities observed across multiple assays.

**TABLE 5:** Antioxidant activities of stem lettuce depending on the presence of peels.

	PSL	USL	*t*
Total polyphenol contents (μg GAE/g)	225.47 ± 0.48	274.08 ± 0.83	−87.495***
Total flavonoids contents (μg QE/g)	184.52 ± 0.33	221.07 ± 0.34	−131.541***
DPPH free radical savenging activity (%)	41.12 ± 0.27	85.89 ± 0.05	−288.042***
ABTS free radical savenging activity (μg Trolox/g)	54.45 ± 1.49	82.77 ± 3.11	−14.222***
Ferric reducing antioxidant power (μg AAE/g)	90.04 ± 0.17	128.00 ± 0.11	−324.458***

*Note:* Values are expressed as mean ± SD (*n* = 3). Statistical significance was determined by independent *t*‐test. Symbols indicate significance: *p* < 0.001 (***).

Abbreviations: PSL, peeled stem lettuce; USL, unpeeled stem lettuce.

#### 3.5.2 Total Flavonoid Content

Table 5 further presents the findings regarding the total flavonoid content in stem lettuce, based on peel treatment. The analysis indicated that the total flavonoid content in USL was 221.072 μg QE/g, whereas PSL recorded a significantly lower value of 184.517 μg QE/g (*p* < 0.05). This observation implies that flavonoid phytochemicals are primarily sequestered within the epidermal tissues. Flavonoids are essential phenolic phytochemicals that confer protection to plants against external threats, including ultraviolet radiation, pathogens, and pests, and are predominantly localized in the epidermis and cortex (Harborne and Williams 2000). The observed reduction in total flavonoid content can be attributed to the excision of these tissues during the peeling process. Indeed, various vegetables, such as lettuce, onions, and potatoes, have been documented to possess higher concentrations of flavonoid phytochemicals in their peels, and the findings of this study corroborate that assertion (Bahorun et al. 2004). Together, these findings confirm that USL contained significantly higher total levels of polyphenols and flavonoids compared to PSL, reinforcing the role of the peel as a major phytochemical reservoir.

#### 3.5.3 DPPH Radical Scavenging Activity

Table 5 delineates the results pertaining to the DPPH radical scavenging activity of stem lettuce, differentiated by peel treatment. The findings reveal that the PSL exhibited a radical scavenging activity of 41.116%, whereas the USL demonstrated a markedly higher activity of 85.888% (*p* < 0.05). Liu et al. (2007) conducted a comparative analysis of DPPH radical scavenging activities across various lettuce cultivars grown in Colorado, USA, and identified red leaf lettuce as having the highest activity (88.9%), followed by butterhead (78.5%), romaine (72.2%), and iceberg (55.1%). The current study indicated that stem lettuce samples retaining peels exhibited antioxidant activity comparable to or exceeding that of some commercial lettuce cultivars.

#### 3.5.4 ABTS Radical Scavenging Activity

The results concerning the ABTS radical scavenging activity of stem lettuce, categorized by peel treatment, are presented in Table 5. The analysis indicates that USL exhibited an ABTS antioxidant activity of 82.786 μg Trolox/g, in contrast to PSL, which demonstrated a significantly lower value of 54.453 μg Trolox/g (*p* < 0.05). These findings suggest that phytochemicals contributing to antioxidant activity are predominantly concentrated in the peel of stem lettuce and that the removal of the peel may result in a decline in the overall antioxidant potential.

#### 3.5.5 FRAP

Table 5 shows the results of the FRAP antioxidant activity assessment of stem lettuce based on peel treatment. The analysis revealed that USL had a FRAP value of 128 μg AAE/g, while PSL recorded a significantly lower value of 90.037 μg AAE/g. Polyphenols and flavonoids are recognized as key antioxidant phytochemicals that substantially influence the FRAP value (Prior et al. 2005), which aligns with the findings for total polyphenol and flavonoid contents in this study. These results indicate that the stem lettuce peel is the primary tissue responsible for the antioxidant capacity. The higher antioxidant capacity observed in USL is likely attributable to the elevated levels of polyphenols and flavonoids in the peel, which are known to act as radical scavengers and reducing agents. Additionally, DPPH and ABTS radical scavenging assays, along with FRAP reducing power tests, demonstrated that USL exhibited significantly greater antioxidant activity than PSL, suggesting a higher concentration of antioxidant phytochemicals in the peel. Moreover, phytochemicals in the peel may act synergistically to enhance their biological effects. Previous studies demonstrated that mixtures of flavonoids and phenolic acids exhibited stronger antioxidant activity than individual compounds alone (Hajimehdipoor et al. 2014), indicating that the combined action of diverse phytochemicals could underlie the pronounced bioactivity of stem lettuce peel. Taken together, the enrichment of polyphenols and flavonoids in USL, along with its higher DPPH, ABTS, and FRAP readouts, provides coherent evidence for a peel‐driven antioxidant mechanism consistent with electron/hydrogen donation and metal‐chelation pathways.

### 3.6 Anti‐Inflammatory Activity

#### 3.6.1 Cell Viability

**FIGURE 2:**
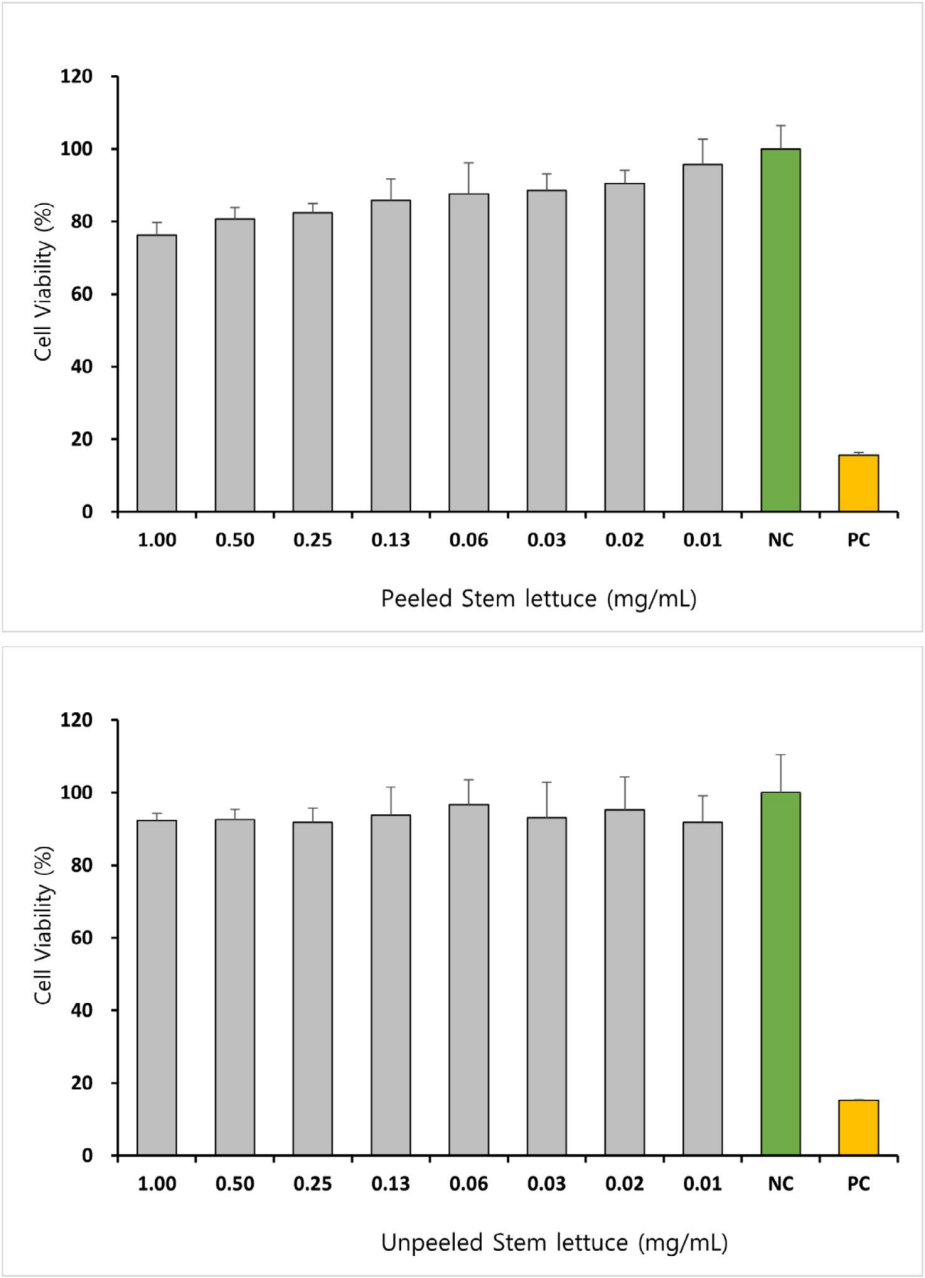
Cell viability of stem lettuce depending on the presence of peels in RAW264.7 cells. Cell viability of RAW264.7 cells after treatment with peeled and unpeeled stem lettuce extracts (0.01–1.00 mg/mL). Data are presented as mean ± SD (*n* = 3). NC (green bar), negative control (untreated cells); PC (yellow bar), positive control (10 μM lactucopicrin).

To assess the cytotoxic effects of stem lettuce extract, a WST‐1 assay was performed on RAW264.7 cells, with the results presented in Figure 2. The PSL and USL samples were evaluated at concentrations ranging from 1.00 to 0.01 mg/mL. A general trend of decreased cell viability was observed with increasing concentrations. PSL maintained cell viability above 75% across all tested concentrations, whereas USL exhibited an even higher viability of over 85%. Both samples demonstrated viability levels comparable to the negative control at concentrations below 0.10 mg/mL, indicating low cytotoxicity. The negative control exhibited approximately 100% viability, whereas the positive control (10 μM lactucopicrin) showed a significant reduction in viability to < 20%, thereby validating the experimental design. Consequently, the final treatment concentrations for subsequent anti‐inflammatory activity assessments were selected to ensure that cell viability remained above 80%. PSL was administered at concentrations of 0.50, 0.10, and 0.05 mg/mL, while USL was administered at 1.00, 0.50, and 0.10 mg/mL.

#### 3.6.2 Inhibition of NO Production

**FIGURE 3:**
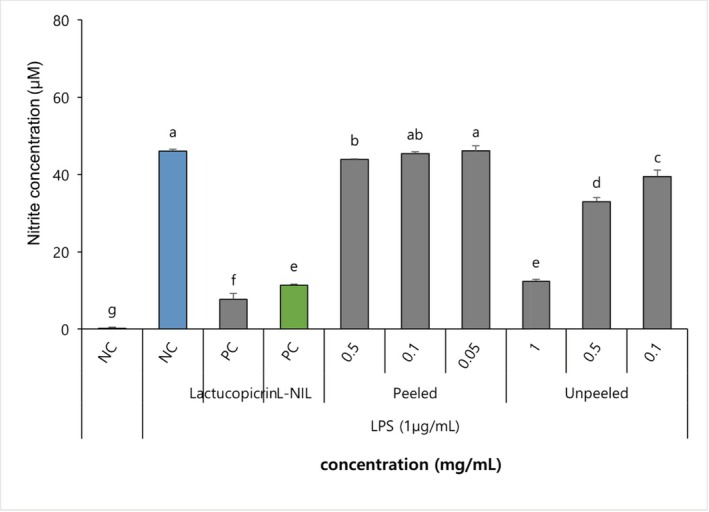
Nitric oxide production of stem lettuce depending on the presence of peels. Nitrite concentration (μM) was measured at 24 h and expressed as mean ± SD (*n* = 3). L‐NIL (green bar), iNOS inhibitor (reference control); NC (blue bar), negative control; PC (gray bar), positive control (LPS only). Different letters above the bars indicate significant differences among groups (*p* < 0.05, one‐way ANOVA followed by Duncan's multiple range test).

The production levels of NO in RAW 264.7 macrophages stimulated with lipopolysaccharide (LPS) at 1 μg/mL are depicted in Figure 3. The negative control exhibited negligible NO production, whereas LPS treatment resulted in a marked increase in NO levels. The positive control, L‐NIL, effectively inhibited NO production, and a significant inhibitory effect was observed in the lactucopicrin‐treated group. The PSL treatment group did not demonstrate significant differences compared to the LPS‐only group at various concentrations, indicating minimal inhibition of NO production. In contrast, the USL treatment group exhibited a gradual decrease in NO production with increasing concentration, demonstrating a strong inhibitory effect comparable to that of lactucopicrin at higher concentrations. At all concentrations, the USL treatment group displayed a greater inhibitory effect than the PSL group, suggesting that USL contains higher concentrations of anti‐inflammatory phytochemicals such as lactucopicrin. In terms of anti‐inflammatory activity, the USL treatment group displayed a concentration‐dependent reduction in NO production and the expression of inflammatory genes (iNOS, COX‐2, TNF‐α, IL‐6, IL‐1β) in LPS‐induced RAW264.7 macrophages, exhibiting markedly superior inhibitory activity compared to the PSL group. This concentration‐dependent NO suppression in USL is consistent with sesquiterpene‐driven interference with pro‐inflammatory signaling and anticipates transcriptional downregulation described below.

#### 3.6.3 Inflammatory Gene Expression

**FIGURE 4:**
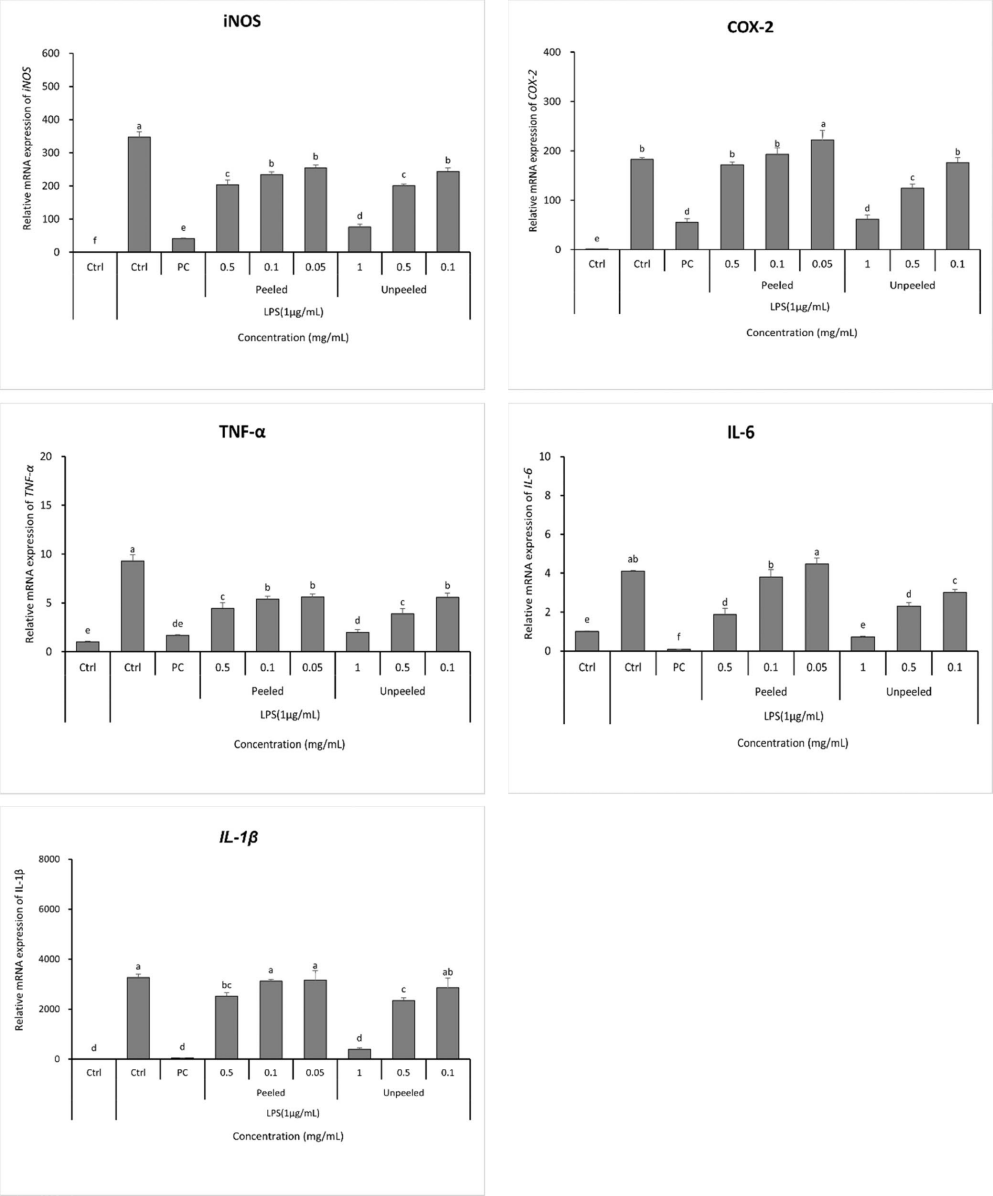
mRNA expression of inflammatory genes by stem lettuce depending on the presence of peels. Relative mRNA levels of iNOS, COX‐2, TNF‐α, IL‐6, and IL‐1β were measured after 24 h and normalized to untreated control (set as 1.0). Data are presented as mean ± SD (*n* = 3). Ctrl, untreated control; PC, positive control (LPS only). Different letters above the bars indicate significant differences among groups (*p* < 0.05, one‐way ANOVA followed by Duncan's multiple range test).

The effects of stem lettuce samples on the expression of inflammatory genes following induction of inflammation are illustrated in Figure 4. In the LPS‐only treatment group, the expression of all inflammation‐related genes, including iNOS, COX‐2, TNF‐α, IL‐6, and IL‐1β, was significantly elevated compared to the negative control, confirming that LPS induces a robust inflammatory response. Conversely, the L‐NIL treatment group, which served as a positive control, exhibited a general reduction in the expression of these genes. When comparing the effects of stem lettuce extracts, the PSL treatment group demonstrated a slight decrease in inflammatory gene expression relative to the LPS group at all concentrations, although the inhibitory effect was limited. Notably, PSL showed minimal inhibitory effects on iNOS and COX‐2 gene expression, indicating its limited anti‐inflammatory activity. In contrast, the USL group showed significantly suppressed gene expression in a concentration‐dependent manner, particularly at 1 mg/mL. A concentration‐dependent inhibitory trend was also observed for TNF‐α and IL‐6, with expression levels decreasing as concentration increased. These findings suggest that specific phytochemicals present in stem lettuce peel, particularly sesquiterpene lactones, may play a role in modulating inflammatory gene expression. The USL treatment group demonstrated inhibitory effects on various inflammatory mediators at the transcriptional level, consistent with previously established results regarding the inhibition of NO production. These findings indicate that sesquiterpene lactones in the peel may interfere with inflammatory signaling pathways such as NF‐κB and MAPK, thereby contributing to the observed reduction in pro‐inflammatory gene expression. These effects are attributed to sesquiterpene lactones abundant in the peel. Lactucopicrin has been demonstrated to inhibit NF‐κB activity in inflammatory macrophages, thereby reducing the production of inflammatory cytokines (He et al. 2021), and to impede importin‐α3‐mediated NF‐κB activity in endothelial cells (Weng et al. 2021). In addition, flavonoids and sesquiterpene lactones may exert anti‐inflammatory effects through multiple pathways beyond NF‐κB inhibition. For instance, flavonoids have been reported to suppress iNOS and COX‐2 expression by blocking NF‐κB and MAPK activation in LPS‐stimulated macrophages (Kwon et al. 2013), suggesting that stem lettuce phytochemicals may act on diverse inflammatory signaling cascades. Although the 1 mg/mL USL group showed slightly lower NO inhibition than the lactucopicrin group, it still demonstrated a significant anti‐inflammatory trend, indicating that the phytochemicals in the peel contributed substantially to the observed bioactivity. However, the in vivo efficacy of these compounds may vary considerably due to limited bioavailability and metabolic transformations. Flavonoids are absorbed in modified forms following intestinal microbiota and hepatic metabolism, indicating that effects observed in vitro may not fully reflect their activity in vivo (Manach et al. 2005). Sesquiterpene lactones predominantly occur as glucuronide or sulfate conjugates after gut microbial and phase II metabolism, which accounts for the low oral bioavailability of lactucopicrin (Weng et al. 2020). Integrating the NO and transcriptional data, the coordinated suppression of iNOS/COX‐2 and key cytokines in USL directly supports inhibition of NF‐κB/MAPK signaling by peel‐derived constituents, providing an explicit mechanism–phenotype link.

## 4 Conclusion

This research involved the application of general component analysis, as well as qualitative and quantitative assessments utilizing UHPLC‐Triple TOF‐ESI MS/MS, to investigate the differences in the physicochemical properties and bioactive components of USL and PSL resulting from the skin treatment of stem lettuce. The study also evaluated antioxidant and anti‐inflammatory activities. Examination of the general components and physicochemical characteristics indicated no significant differences in moisture, salinity, or soluble solid content attributable to peel treatment (*p* > 0.05). This study demonstrated that the peel of stem lettuce is rich in phytochemicals with significant antioxidant and anti‐inflammatory properties, underscoring its potential as a valuable functional material derived from food byproducts. Therefore, this study suggests that stem lettuce peel exhibits antioxidant and anti‐inflammatory activities, supporting its potential as a functional material. Further validation through animal experiments and clinical trials is required to substantiate its relevance to human health. In summary, stem lettuce peel represents a promising upcycled ingredient; future work should confirm efficacy in vivo and address formulation, stability, and bioavailability to enable translation into functional foods and nutraceuticals. Beyond health functionality, valorization of stem lettuce peel also contributes to sustainability goals by reducing agricultural waste and promoting circular economy approaches in the food industry. Nevertheless, limitations remain: the present study relied on in vitro assays only, and results may differ under physiological conditions. Furthermore, sensory properties and processing stability of peel‐derived ingredients need to be evaluated before industrial application. Overall, these findings provide a foundation for the development of peel‐based functional ingredients, while also highlighting the need for integrative research spanning biochemical, technological, and clinical domains.

## Author Contributions

**Sun Young Han:** conceptualization (lead), data curation (lead), formal analysis (equal), methodology (equal), writing – original draft (lead), writing – review and editing (equal). **Ju Hong Park:** funding acquisition (lead), project administration (lead), supervision (equal), visualization (lead). **Nami Joo:** investigation (equal), resources (lead), software (equal), validation (equal).

## Ethics Statement

The authors have nothing to report.

## Conflicts of Interest

The authors declare no conflicts of interest.

## Data Availability

The data that support the findings of this study are available from the corresponding author upon reasonable request.
